# The Genetic Background of the *Curly Tail* Strain Confers Susceptibility to Folate-Deficiency-Induced Exencephaly

**DOI:** 10.1002/bdra.20632

**Published:** 2009-10-12

**Authors:** Katie A Burren, John M Scott, Andrew J Copp, Nicholas D E Greene

**Affiliations:** 1 Neural Development Unit, UCL Institute of Child HealthLondon, UK; 2 School of Biochemistry and Immunology, Trinity College DublinIreland

**Keywords:** neural tube defects, folic acid, inositol, exencephaly, curly tail, diet

## Abstract

**BACKGROUND**: Suboptimal maternal folate status is considered a risk factor for neural tube defects (NTDs). However, the relationship between dietary folate status and risk of NTDs appears complex, as experimentally induced folate deficiency is insufficient to cause NTDs in nonmutant mice. In contrast, folate deficiency can exacerbate the effect of an NTD-causing mutation, as in *splotch* mice. The purpose of the present study was to determine whether folate deficiency can induce NTDs in mice with a permissive genetic background which do not normally exhibit defects. **METHODS**: Folate deficiency was induced in *curly tail* and genetically matched wild-type mice, and we analyzed the effect on maternal folate status, embryonic growth and development, and frequency of NTDs. **RESULTS**: Folate-deficient diets resulted in reduced maternal blood folate, elevated homocysteine, and a diminished embryonic folate content. Folate deficiency had a deleterious effect on reproductive success, resulting in smaller litter sizes and an increased rate of resorption. Notably, folate deficiency caused a similar-sized, statistically significant increase in the frequency of cranial NTDs among both *curly tail* (*Grhl3* mutant) embryos and background-matched embryos that are wild type for *Grhl3*. The latter do not exhibit NTDs under normal dietary conditions. Maternal supplementation with *myo*-inositol reduced the incidence of NTDs in the folate-deficient wild-type strain. **CONCLUSIONS**: Dietary folate deficiency can induce cranial NTDs in nonmutant mice with a permissive genetic background, a situation that likely parallels gene-nutrient interactions in human NTDs. Our findings suggest that inositol supplementation may ameliorate NTDs resulting from insufficient dietary folate. Birth Defects Research (Part A), 2010. © 2009 Wiley-Liss, Inc.

## INTRODUCTION

Maternal nutritional status is thought to play a key role in embryonic development and determination of pregnancy outcome. In particular, attention has focused on folic acid, a vitamin whose biologic derivatives, folates, are integral to the interlinked folate and methionine cycles that make up one-carbon metabolism (Blom et al.,^2006^; Beaudin and Stover^2009^). Maternal folic acid supplementation during early pregnancy reduces the risk of neural tube defects (NTDs; see Blom et al.,^2006^ and Molloy et al.,^2009^ for reviews). Spina bifida and anencephaly, the most frequent NTDs, result from failure of closure of the neural tube during embryogenesis and are among the commonest human birth defects (Copp et al.,^2003^; Greene and Copp,^2009^). Additional evidence for the potential importance of adequate folate one-carbon metabolism in neural tube closure comes from the finding that indicators of suboptimal folate status, such as low serum folate levels or elevated plasma homocysteine, are risk factors for NTDs (Kirke et al.,^1993^; Mills et al.,^1995^). Polymorphisms in certain enzymes of folate one-carbon metabolism are associated with increased risk of NTDs in certain populations (Boyles et al.,^2005^; Molloy et al.,^2009^). Moreover, some NTD cases may be associated with an underlying defect in folate metabolism in the fetus itself (Dunlevy et al.,^2007^).

Maternal folate levels in most NTD-affected pregnancies do not correspond to overt folate deficiency and lie within what is considered the ‘normal’ range (Scott,^1999^). However, there is an apparent relationship between maternal folate status and NTD risk (Daly et al.,^1995^; Shaw et al.,^1995^), suggesting that low folate status may increase susceptibility. Possible effects of limited folate availability on neural tube closure have been investigated experimentally in the mouse. The major sources of folates are dietary (food folates and synthetic folic acid supplements) and from bacterial microflora of the large intestine (Rong et al.,^1991^; Beaudin and Stover,^2009^). Therefore, induction of severe folate deficiency in experimental models requires the use of a folate-deficient diet in combination with antibiotics (Burgoon et al.,^2002^; Burren et al.,^2008^). Imposition of maternal folate deficiency in ‘normal’ inbred mouse strains, Swiss-Webster and ICR, resulted in growth retardation and reduced weight of offspring, as well as an increased rate of resorption (Heid et al.,^1992^; Burgoon et al.,^2002^). However, NTDs were not observed in these studies.

The finding of overlapping maternal folate levels between normal and NTD pregnancies in humans, and the inability of severe folate deficiency to cause NTDs in nonmutant mice, argues against the idea that folate deficiency alone is a major cause of NTDs. Instead, it is hypothesized that a proportion of NTDs may result from a gene-environment interaction, in which suboptimal folate status exacerbates an underlying genetic predisposition. This idea received support from recent studies in which maternal folate deficiency was found to increase the rate of cranial NTDs in *Pax3* (*splotch; Sp*^*2H*^) mutant embryos (Burren et al.,^2008^; Greene et al.,^2009^). In contrast, wild-type littermates did not display NTDs when folate deficient, suggesting that NTDs in the mutant embryos resulted from an interaction of folate deficiency with NTD predisposition, imposed by loss of Pax3 function. In addition to an exacerbating effect of folate deficiency, NTDs in *splotch* embryos developing under normal ‘folate-replete’ conditions may be further ameliorated by supplemental folic acid (Fleming and Copp,^1998^; Wlodarczyk et al.,^2006^).

The increased penetrance of cranial NTDs in *Pax3* mutant embryos demonstrates that folate deficiency can increase susceptibility to NTDs. However, rather than involving a single gene, the majority of human NTDs are thought to result from the interaction of multiple predisposing genetic factors, plus a possible contribution of environmental factors (Harris and Juriloff,^2007^). Therefore, to more accurately model this etiology, we decided to test whether folate deficiency is sufficient to induce NTDs in a mouse strain in which NTDs never arise under normal dietary conditions, but which carries ‘susceptibility’ modifier genes proven to enhance NTDs in the context of a major genetic mutation.

For this purpose we used a wild-type mouse strain, denoted +^*ct*^/+^*ct*^, that shares the genetic background of the *curly tail* (*ct/ct*) NTD mutant strain. Homozygous *curly tail* (*ct/ct*) embryos develop spina bifida (approximately 15–20% penetrance), tail flexion defects (approximately 50%), and infrequent exencephaly (Van Straaten and Copp,^2001^). The *curly tail* gene, *ct*, corresponds to a hypomorphic allele of the transcription factor *grainyhead-like-3* (*Grhl3*) (Ting et al.,^2003^; Gustavsson et al.,^2007^). However, genetic modifiers in the *curly tail* strain also have a major effect on penetrance of spinal NTDs (Neumann et al.,^1994^; Letts et al.,^1995^; Gustavsson et al.,^2008^). We previously generated the genetically matched, partially congenic, wild-type strain (+^*ct*^/+^*ct*^) by successive back-crossing to the *ct* strain, having first replaced the *ct* allele of *Grhl3* with a wild-type allele. The +^*ct*^/+^*ct*^ strain is predicted to share 96% of the *curly tail* genetic background (Van Straaten andCopp,^2001^; Gustavsson et al.,^2007^). Embryos of the +^*ct*^/+^*ct*^ strain do not develop NTDs or tail flexion defects, but, as they carry modifier genes from the *curly tail* background, we reasoned that embryos may exhibit predisposition to NTDs caused by an environmental insult such as folate deficiency.

Although spinal NTDs in the *curly tail* mutant are amenable to prevention by certain exogenous agents, including inositol and retinoids, there is no protective effect of folic acid, and *curly tail* has therefore been used as a model for ‘folate-resistant’ NTDs (Seller,^1994^; Chen et al.,^1994^; Greene and Copp,^1997^,^2005^). However, the effect of folate deficiency has not been investigated in *curly tail,* and the present study provided an opportunity to ask whether folate deficiency can exacerbate NTDs, in a mutant mouse strain in which spinal NTDs are not preventable by supplemental folic acid (folate resistant). Our findings demonstrate a deleterious effect of dietary folate deficiency on cranial neural tube closure, with an enhanced frequency of exencephaly in both *curly tail* mutant embryos and the congenic wild-type (+^*ct*^/+^*ct*^) strain in which NTDs never normally occur.

## MATERIALS AND METHODS

### Maintenance of Mice and Dietary Conditions

*Curly tail* (*ct/ct*) and genetically matched wild-type (+^*ct*^/+^*ct*^) mice were maintained as random-bred, homozygous colonies. Mice were routinely fed a standard breeding diet (Harlan Teklad) containing 2.7 mg/kg folic acid. For dietary studies, female mice were randomly allocated at 6 weeks of age to the standard diet (SD) or to one of three synthetic protein-based diets that were matched to the standard diet for micronutrient levels, with the exception of folic acid, as used previously (Burren et al.,^2008^). No folic acid was present in diet FD (folate deficient), which also contained the antibiotic 1% succinyl sulfathiazole (10 gm/kg) to produce the most profound folate deficiency. Two control diets were FD+BF (folate deficient with bacterially derived folate), which lacked folate but with omission of antibiotic, and FD+DF (folate deficient with dietary folate), which contained the same level of folic acid as the standard breeding diet, but with the addition of antibiotic. Females were maintained on each diet for a minimum of four weeks before mating to SD-fed males.

### Supplementation of Experimental Litters and Embryo Analysis

Experimental litters were generated by overnight matings, the day of finding a copulation plug being designated embryonic day 0.5 (E0.5). In vivo supplementation was by intra-peritoneal injection, daily from E7.5–10.5, using 1 mg/ml folic acid (Sigma) in sterile phosphate buffered saline (PBS), to a dose of 10 mg/kg, or 40 mg/ml *myo*-inositol (Sigma) in PBS, to a dose of 400 mg/kg. Pregnant females were killed by cervical dislocation. Embryos were dissected from the uterus in Dulbecco's Modified Eagle's Medium (Invitrogen) containing 10% fetal calf serum, and inspected carefully to detect any abnormalities of neural tube closure. Somite number and crown-rump length were determined as described previously (Brown,^1990^). Embryos were then rinsed in PBS and immediately frozen on dry ice for biochemical analysis.

### Quantification of Folate and Homocysteine in Maternal Blood and Embryos

Mice were anesthetized with halothane, and blood was collected by cardiac puncture and immediately transferred to lithium-heparin coated tubes (Microtainer, BD). Whole blood folate was measured using the *L. casei* microbiologic method (Molloy and Scott,^1997^). A second aliquot was centrifuged, and plasma was stored at −70°C before quantification of homocysteine by standard methods (Leino,^1999^). Embryonic folate was measured using an adapted version of the *L. casei* microbiologic method (Molloy and Scott,^1997^), as described previously (Burren et al.,^2008^).

### Statistical Analysis

Comparisons were performed between treatment groups by ANOVA, *t* test (for pairwise comparisons), or *z*-test (comparison of NTD frequencies), computed using Sigmastat (version 3.5, Systat Software) and/or SPSS (version 13).

## RESULTS

### Folate-Deficient Diets Lower Maternal Blood Folate and Elevate Homocysteine

**Figure 1:**
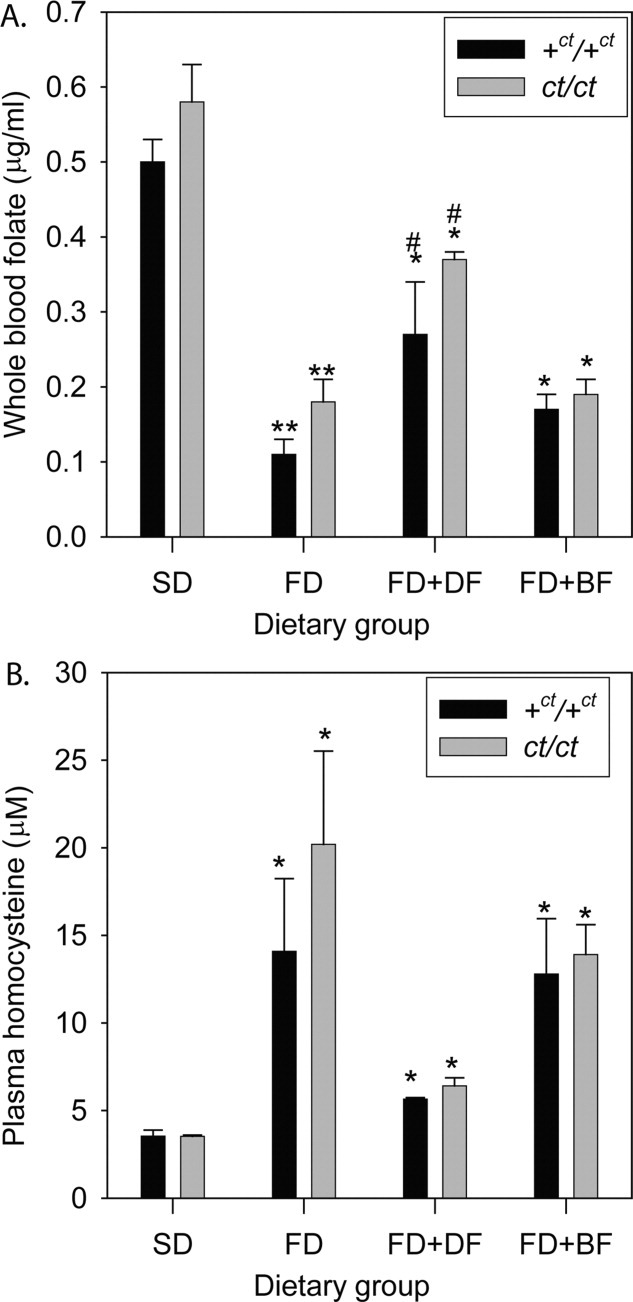
Effect of dietary folate deficiency on maternal blood folate (**A**) and plasma homocysteine (**B**). Folate-deficient (FD) conditions, imposed by a folate-free diet containing antibiotic, resulted in a significant reduction in maternal whole blood folate compared to standard diet (SD) and a significant increase in maternal plasma homocysteine. Inclusion of either dietary folate (FD+DF) or omission of antibiotic (FD+BF) had an intermediate effect on both maternal folate and homocysteine level. Significant differences compared to SD are indicated by * (*p* < 0.05) or ** (*p* < 0.001). Significant difference compared to FD diet is indicated by # (*p* < 0.05). No significant differences were observed between the +^*ct*^/+^*ct*^ and *ct/*ct strains for any of the dietary conditions.

Dietary folate deficiency was induced in wild-type (+^*ct*^/+^*ct*^) and *curly tail* (*ct/ct*) female mice, by maintenance on a synthetic folate-free diet including antibiotics (designated FD) for a minimum of four weeks before mating. As in our previous study of *splotch* mice (Burren et al.,^2008^), these conditions resulted in a significant reduction in maternal whole blood folate level, compared to a standard breeding diet (SD) (Fig. 1A). A control diet (FD+DF) that included folic acid, but with antibiotic to remove bacterial folate, resulted in a less severe reduction in maternal blood folate levels. In contrast, omission of dietary folate, without addition of antibiotics (FD+BF), had an effect of similar magnitude as the FD diet (Fig. 1A).

In parallel with reduction of maternal blood folate, the FD diet also resulted in a significant elevation of maternal homocysteine (Fig. 1B). Homocysteine was also significantly elevated, but to a lesser extent, in mice maintained on the FD+DF and FD+BF diets, in which either bacterial folate or dietary folate were omitted (Fig. 1B).

### Decreased Reproductive Performance in Folate-Deficient Pregnancies

Previously, using the same dietary conditions, we found no effect of folate deficiency on litter size or resorption rate in *Pax3* mutant *splotch* (*Sp*^*2H*^) mice (Burren et al.,^2008^). In contrast, folate deficiency had a deleterious effect on reproductive success in +^*ct*^/+^*ct*^ and *curly tail* pregnancy. Among litters analyzed at E11.5, folate deficiency resulted in a significant reduction in the number of implantations per litter and an increase in the number of resorptions (Table 1). This sensitivity to folate status seems to be a feature of the *ct* genetic background because litters of both +^*ct*^/+^*ct*^ and *ct/ct* strains appeared equally affected. The deleterious effects of folate deficiency on embryonic viability appeared to worsen progressively during development because, among litters collected at E13.5 (n = 5 +^*ct*^/+^*ct*^ and 6 *ct/ct*), very few viable embryos were observed, whereas the resorption rate was very high (5.5 ± 0.6 and 5.8 ± 1.3 per litter, respectively).

**Table 1:** Impact of Folate-Deficient Diets on Litter Size and Resorption Rate

Strain	Diet	No. litters	Total implants	Implants per litter	Resorptions per litter
+^*ct*^/+^*ct*^	SD	15	102	6.8 ± 0.8	0.7 ± 0.3
	FD	14	68	4.9 ± 0.7	2.0 ± 0.7*
	FD+DF	4	21	5.3 ± 0.7	1.0 ± 0.2
	FD+BF	4	23	5.8 ± 0.3	2.0 ± 0.3*
*ct/ct*	SD	17	123	7.2 ± 0.1	0.9 ± 0.2
	FD	20	106	5.3 ± 0.5*	2.3 ± 0.4*
	FD+DF	7	47	6.7 ± 0.3	1.1 ± 0.2#
	FD+BF	7	46	6.6 ± 0.3	1.9 ± 0.2*

Total number of implantations and number of resorptions was recorded for litters collected at E11.5. Values are given as mean ± SEM. FD conditions resulted in smaller litter size and increased resorption rate (* indicates significant difference from SD, *p* < 0.05, ANOVA). The inclusion of dietary folate (FD+DF) reduced the resorption rate (# significant difference compared with FD, *p* < 0.05), whereas omission of antibiotic did not (* significant difference from SD, *p* < 0.05). No significant differences were observed between +^*ct*^/+^*ct*^ and *ct/ct* litters for any parameter.

**Figure 2:**
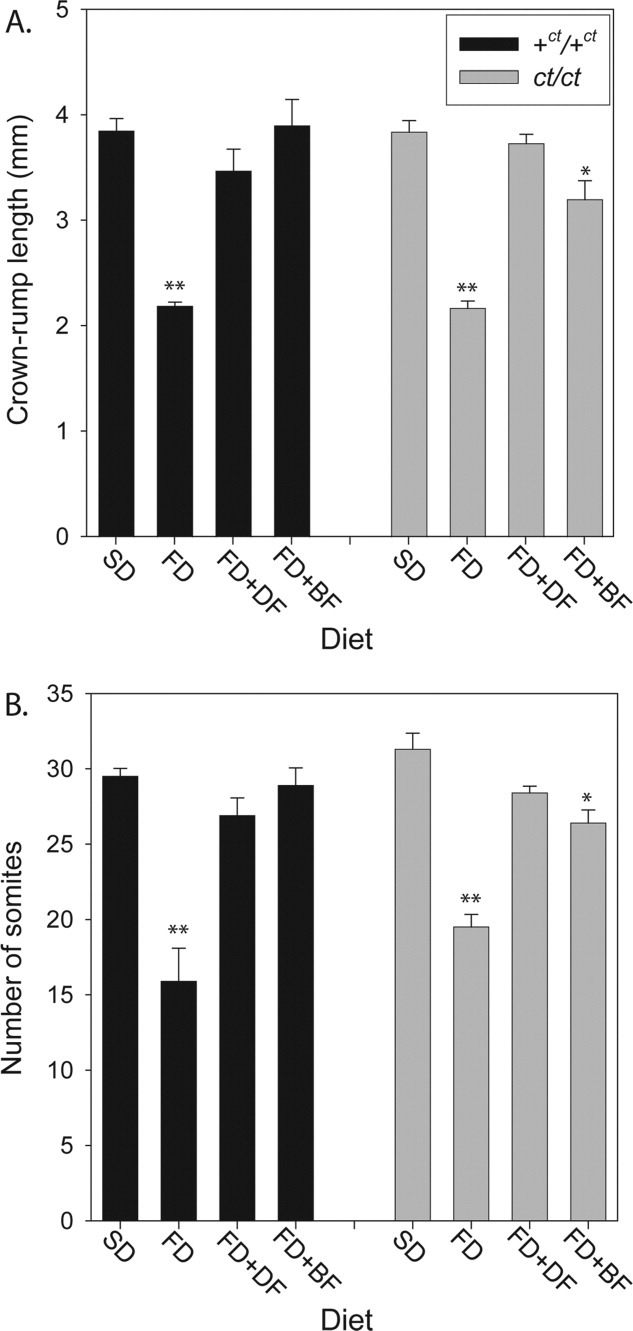
Dietary folate deficiency causes embryonic growth retardation. The growth and developmental progression of +^*ct*^/+^*ct*^ and *ct/ct* embryos were assessed at E10.5 by analysis of (**A**) crown-rump length and (**B**) somite number. The folate-deficient diet (FD) resulted in a significant reduction in both parameters (**p < 0.02, compared to all other groups of same genotype). Inclusion of either dietary folate (FD+DF) or bacterial folate (FD+BF) improved both growth and developmental progression. However, crown-rump length and number of somites remained significantly lower for embryos on the FD+BF diet compared to standard (SD) conditions (*p < 0.02). No significant differences were observed between +^*ct*^/+^*ct*^ and *ct/ct* strains for any of the dietary conditions.

To assess growth and developmental progression, we determined crown-rump length and number of somites, respectively, of embryos at E10.5 that had developed under differing dietary conditions. Compared to the standard diet, folate deficiency resulted in a significant reduction in crown-rump length and number of somites in both +^*ct*^/+^*ct*^ and *ct/ct* strains (Fig. 2). Thus, FD conditions appeared to dissociate gestational age from embryonic growth and developmental progression such that, by E10.5, the size and somite stage of FD embryos corresponded to approximately 24 hours developmental delay compared to standard conditions. Inclusion of dietary folate or omission of antibiotics (FD+DF and FD+BF) significantly enhanced growth parameters compared to FD conditions (Fig. 2).

### Embryonic Folate Content, but Not Folate Concentration, Is Diminished in Folate Deficiency

Somite-matched +^*ct*^/+^*ct*^ and *ct*/*ct* embryos were analyzed to determine the effect of FD conditions on embryonic folate content and concentration. The total folate content of 26–32 somite FD embryos was significantly diminished compared to embryos that developed under standard conditions (Table 2), despite the fact that FD embryos were gestationally older than SD embryos at the same somite stage. In contrast, folate concentration (normalized to protein content of the embryo) did not differ significantly between dietary groups because protein content was also lower in FD conditions. These findings suggest that folate availability places a limit on growth and developmental progression during mouse embryogenesis.

**2:** Embryonic Folate Content and Concentration in +^*ct*^/+^*ct*^ and *ct*/*ct* Embryos

Strain	Diet	No. embryos	No. somites Mean (range)	Folate content (pg/embryo)	Folate concentration (pg/mg protein)
+^*ct*^/+^*ct*^	SD	18	29.8 (26–32)	8244 ± 445	30.1 ± 3.3
	FD	13	28.0 (26–32)	5184 ± 282*	37.3 ± 1.3
	FD+FA	5	28.8 (26–32)	13,675 ± 598^#^*	49.0 ± 9.2*
*ct/ct*	SD	10	28.4 (26–32)	8822 ± 1527	28.8 ± 3.2
	FD	14	29.1 (26–32)	5065 ± 419*	23.4 ± 5.6
	FD+FA	7	28.1 (26–32)	8877 ± 975^#^	56.0 ± 7.9^#^

Total folate content (mono- and polyglutamates) was determined in embryos from the standard diet (SD) and folate-deficiency (FD) groups that had reached an equivalent developmental stage (somite-stage matched). Values are given as mean ± SEM. Folate content did not differ between genotypes but was significantly diminished in FD conditions (*p < 0.05 compared with SD). Folate concentration did not differ either between genotypes or dietary groups. Folic acid treatment (FD+FA) resulted in a significant increase in folate content compared with untreated folate-deficient embryos (#*p* < 0.05 compared with FD).

### Folate Deficiency Causes Cranial NTDs in +^*ct*^/+^*ct*^ and *ct/ct* Embryos

**Figure 3:**
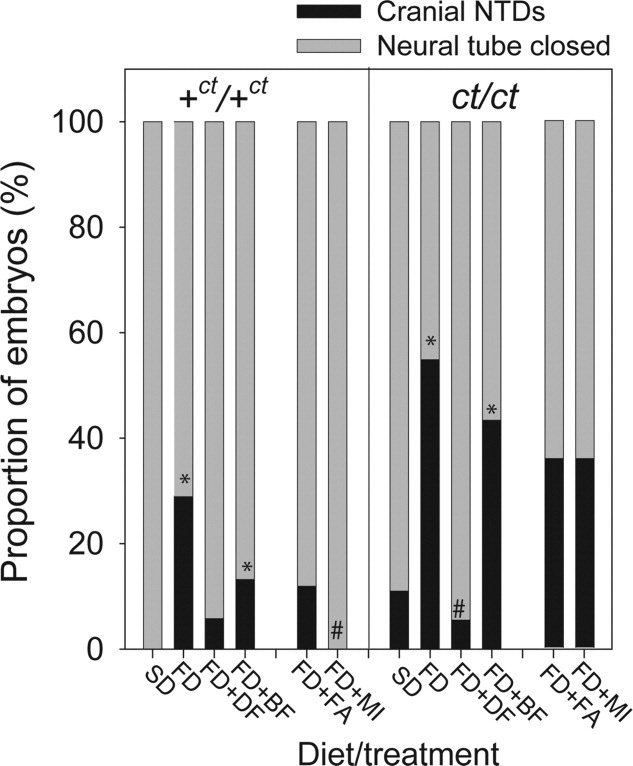
Folate deficiency increases the risk of cranial NTDs among +^*ct*^/+^*ct*^ and *ct*/*ct* embryos. Development on the folate-deficient (FD) diet resulted in an increased frequency of cranial NTDs among both +^*ct*^/+^*ct*^ and *ct/ct* embryos (* indicates significant difference compared to SD, *p* < 0.001). Inclusion of folic acid in the diet (FD+DF) reduced the incidence of cranial NTDs (# significant difference compared to FD, *p* < 0.001), whereas removal of antibiotic (FD+BF) had a lesser, nonsignificant effect. The frequency of cranial NTDs appeared lower in embryos following maternal supplementation with folic acid (FD+FA) or *myo*-inositol (FD+MI), although only the protective effect of *myo*-inositol in +^*ct*^/+^*ct*^ embryos was statistically significant (# compared to FD, *p* < 0.02). Number of embryos in which cranial neural tube closure was assessed, in order of x-axis group: +^*ct*^/+^*ct*^ = 102, 48, 17, 15, 17, 26; *ct/ct* = 126, 92, 36, 23, 23, 22.

We examined embryos at E11.5 to determine whether neural tube closure was compromised by development under FD conditions. Remarkably, we observed cranial NTDs (exencephaly) among +^*ct*^/+^*ct*^ embryos, whereas this strain has never been found to develop NTDs under normal dietary conditions, either in this (Fig. 3) or in previous studies. A lower frequency of cranial NTDs was also observed among +^*ct*^/+^*ct*^ embryos developing under FD+DF or FD+BF conditions. The order of diets in terms of increasing incidence of NTDs—from SD (no NTDs), FD+DF, FD+BF, to FD (highest frequency)—correlates with the order of decline in maternal blood folate levels. Spinal NTDs were not observed among +^*ct*^/+^*ct*^ embryos (n = 15 FD embryos at stages at which the spinal neural tube is expected to be closed).

*Curly tail* (*ct/ct*) mutant embryos displayed a low frequency (11%) of cranial NTDs under normal dietary conditions (Fig. 3), as reported previously (Van Straaten and Copp^2001^). However, folate deficiency caused a dramatic increase in the frequency of cranial NTDs in *ct/ct* embryos to more than 50% (Fig. 3). This effect was not observed when folic acid was included in the diet (FD+DF), correlating with an elevation of maternal blood folate (Fig. 1A). In contrast, cranial NTDs still occurred at high frequency when bacterial folate was available because of omission of antibiotic (Fig. 3; FD+BF), again correlating with an apparent lack of difference between FD and FD+BF conditions in effect on maternal blood folate (Fig. 1A). Although many embryos were developmentally delayed we could assess posterior neuropore closure among embryos that had 32 or more somites. Spina bifida was observed in 6 of 21 (28.6%) folate-deficient *ct/ct* embryos, which does not differ significantly from the typical 20% frequency that was observed among *curly tail* embryos developing under standard dietary conditions.

A comparison of the effect of FD diet on the different strains showed that exencephaly was significantly more frequent among FD *ct/ct* embryos than +^*ct*^/+^*ct*^ (55% compared to 29%; *p* < 0.01). However, taking into consideration the baseline 11% exencephaly rate among *ct/ct* embryos, observed even under SD conditions, there was no significant difference between strains in the increase in NTD frequency attributable to folate deficiency.

Finally, we tested whether maternal supplementation with either folic acid or *myo*-inositol would reduce the detrimental effects of dietary folate deficiency. In +^*ct*^/+^*ct*^ embryos, treatment of pregnant dams with *myo*-inositol abolished cranial NTDs (*p* < 0.02; Fig. 3). Folic acid supplementation caused a statistically nonsignificant reduction in frequency, concomitant with an increase in embryonic folate content (Table 2). The frequency of cranial NTDs in *ct/ct* mutants was lower when dams were supplemented with folic acid or *myo*-inositol (Fig. 3), but neither effect reached statistical significance.

## DISCUSSION

In mammals, folates cannot be synthesized de novo and must be obtained from exogenous sources via intestinal absorption. Polyglutamated folates are converted to monoglutamates for absorption and circulate in the serum predominantly as 5-methyl-tetrahydrofolate. Cellular folate appears essential for embryonic survival and neural tube closure because a proportion of embryos lacking folate receptor 1 (Folr1) develop NTDs, when rescued from lethality by administration of folinic acid (Piedrahita et al.,^1999^; Spiegelstein et al.,^2004^). Nevertheless, elucidation of a link between dietary folate deficiency and risk of NTDs has proven more elusive.

In the present study, maintenance of female +^*ct*^/+^*ct*^ or *ct*/*ct* mice on a folate-deficient diet, containing antibiotic to deplete the intestinal microflora, resulted in reduced blood folate and elevated plasma homocysteine, confirming previous findings of ourselves and others (Burgoon et al.,^2002^; Burren et al.,^2008^). This experimental model recapitulates features associated with suboptimal dietary folate status in human NTDs. However, despite causing severe folate deficiency and embryonic growth retardation, NTDs have never been induced in nonmutant strains (Heid et al.,^1992^; Burgoon et al.,^2002^; Burren et al.,^2008^).

It was particularly striking, therefore, that in the present study folate deficiency proved sufficient to cause cranial NTDs in +^*ct*^/+^*ct*^ embryos, a nonmutant strain that does not exhibit NTDs under standard dietary conditions. Previously we observed an enhanced frequency of cranial NTDs in *splotch* embryos, both homozygous (*Sp^2H^/Sp^2H^*) and heterozygous (*Sp^2H^/+*), developing under folate-deficient conditions (Burren et al.,^2008^). Similarly, in the SELH/Bc mouse strain, risk of cranial NTDs is determined not only by a combination of genes at three different loci, but also by diet (although not by folate level) (Juriloff et al.,^2001^; Harris and Juriloff,^2005^). Therefore, we hypothesize that folate-related NTDs in +^*ct*^/+^*ct*^ embryos result, as in *splotch* and SELH/Bc, from gene-environment interactions. The difference in the case of +^*ct*^/+^*ct*^embryos, however, is that the genetic risk factors are insufficient to cause NTDs by themselves. This latter etiology may well parallel the situation in humans in which suboptimal folate status (or other dietary factors) enhances susceptibility to NTDs when present in combination with genetic risk factors that may not be causative under folate-replete conditions.

Although +^*ct*^/+^*ct*^ embryos are wild type for *Grhl3*, the major *curly tail* gene, predisposing modifiers are known to be present in the *curly tail* genetic background, with at least three risk loci for spinal NTDs having been mapped (Neumann et al.,^1994^; Letts et al.,^1995^). Findings of the current study indicate that the *ct* strain genetic background also contains susceptibility loci for folate-related cranial NTDs. It is plausible that a common set of genetic risk factors may influence both cranial and spinal neural tube closure in *curly tail* mice, because exencephaly and spina bifida occur together in a number of mouse mutants (Copp et al.,^2003^). However, at present it is not known whether any or all of the modifiers that influence penetrance of spinal NTDs in *curly tail* mice are the same as those that are permissive for folate-deficiency-induced exencephaly. The effect of dietary folate deficiency on neural tube closure has been investigated in several mouse strains, including Swiss-Webster (Heid et al.,^1992^), ICR (Burgoon et al.,^2002^), *Sp* wild-type (C57BL/6 background; Li et al.,^2006^), Sp^2H^ wild-type (mixed CBA/101 and C3H background; Burren et al.,^2008^), and CD1 (our unpublished data). Although folate deficiency does not cause NTDs in any of these strains, it is possible that the folate-sensitivity variants in the *ct* genetic background (mixed CBA/Gr and GFF) will prove to be present in other as yet untested strains.

It is informative to compare the predisposition to cranial NTDs in the different *curly tail* (*Grhl3*)-related strains that have been described to date. Two independent *Grhl3* gene knockouts exist, which exhibit 2% and 14% exencephaly, respectively (Ting et al.,^2003^; Yu et al.,^2006^). NTDs in the former strain are resistant to folic acid supplementation, whereas the effect of folate deficiency on *Grhl3* null embryos has not been reported. In our studies the *ct/ct* mutant, which is a hypomorphic allele of *Grhl3,* exhibited a very similar (11%) frequency of cranial NTDs as the gene knockouts, but with an exacerbation to 55% when folate deficient. Moreover, the +^*ct*^/+^*ct*^ congenic strain, which is wild type at the *Grhl3* locus but incorporates essentially all of the *curly tail* genetic background modifiers, developed no cranial NTDs under folate-replete conditions, but exhibited exencephaly in 29% of cases when folate deficient. Given this comparison, it is tempting to attribute the great majority of the predisposition to cranial NTDs to the genetic background effect, although having a loss-of-function *Grhl3* allele clearly imparts a minor risk of cranial NTD.

The occurrence of spinal NTDs (open spina bifida) provides a sharp contrast to the situation with cranial NTDs. Both of the *Grhl3* gene knockout strains exhibit 100% spina bifida (Ting et al.,^2003^; Yu et al.,^2006^), whereas the hypomorphic *ct/ct* strain typically shows 15–20% spina bifida, and the +^*ct*^/+^*ct*^ congenic strain does not exhibit spinal NTDs at all. Although the limited period of embryo viability hampered our analysis of spinal neural tube closure in folate-deficient litters, there appeared to be no exacerbation of spina bifida by folate deficiency in the *ct/ct* strain nor in folate-deficient +^*ct*^/+^*ct*^ litters, where spina bifida was never observed. Hence, the risk of spinal NTDs appears to be predominantly determined by the degree of loss of Grhl3 function. Differential regulation of NTDs in the cranial and spinal regions is also observed in the very different response of *curly tail* mice to retinoic acid (RA) treatment. Exogenous RA was found to exacerbate cranial NTDs when administered on day 8, the critical stage for cranial neurulation, whereas spinal NTD frequency was reduced by RA on day 9, the key stage for spinal closure (Seller et al.,^1979^; Seller and Perkins,^1982^; Chen et al.,^1994^). It now seems likely that RA may interact with cranial and spinal closure via two different embryonic mechanisms that have distinct genetic regulatory requirements.

In a previous study, a low-folate diet had no effect on NTD frequency in *ct/ct* embryos (Tran et al.,^2002^). However, the diets used in that study contained minimal levels of folic acid (0.3 mg/kg) and no antibiotics, suggesting that residual folate may have been available. The lack of effect on NTD incidence thus correlates with our finding that availability of even low levels of dietary folate appears sufficient to enable neural tube closure and dramatically enhance embryonic folate content (current study and Burren et al.,^2008^).

In addition to impaired cranial neurulation, there was a significant decrease in litter size and an increase in resorption rate in the FD diet group, suggesting that folate deficiency results in embryonic lethality. This finding differs from our previous study in which there was no effect on litter size, using the same dietary model in *splotch* mice (Burren et al.,^2008^). This observation suggests that the *curly tail* genetic background confers a particularly high sensitivity to folate-deficient conditions, which appears to reflect a requirement of the embryo because maternal blood folate levels in *ct/ct* and +^*ct*^/+^*ct*^mice are affected by the FD diet to a similar extent as other strains (unpublished observations). The reduced survival of folate-deficient *ct/ct* and +^*ct*^/+^*ct*^ embryos may reflect the severe growth retardation and developmental delay that was observed among surviving embryos. Although most embryos analyzed at E10.5–11.5 had reached a developmental stage (judged by somite number) at which the cranial neural folds should have been closed, the developmental delay suggests that the process of cranial neurulation would have occurred over a longer period. We hypothesize that cranial NTDs in folate-deficient conditions occur as a result of reduced proliferation associated with growth retardation. In support of this idea, the incidence of cranial NTDs in *ct/ct* embryos was previously found to be enhanced by treatment with inhibitors of DNA synthesis such as hydroxyurea, mitomycin C, or 5-fluorouracil (Seller and Perkins,^1983^,^1986^).

Much of the effect of the FD diet on neural tube closure in this study appeared to be due to lack of dietary folate as opposed to removal of gut bacteria, because inclusion of folic acid in the diet was sufficient to virtually eliminate NTDs, even when antibiotics were included. Maternal supplementation with folic acid by intra-peritoneal injection produced a small but nonsignificant reduction in the incidence of exencephaly and, hence, was less effective than inclusion of folic acid in the diet throughout gestation. It is possible that embryos may already have been developmentally retarded by E7.5, at which point initiation of folic acid treatment was insufficient to normalize development. Remarkably, *myo*-inositol supplementation was able to prevent folate-related NTDs in +^*ct*^/+^*ct*^ embryos, although not significantly in *ct/ct* embryos. Inositol has previously been found to prevent spinal NTDs in *curly tail* mice, whereas FA has no protective effect (Greene and Copp,^1997^; Cogram et al.,^2004^). A requirement for inositol in cranial neurulation has also been demonstrated: inositol deficiency causes cranial NTDs in mice, even among nonmutant strains (Cockroft et al.,^1992^). Inositol supplementation also has a protective effect against diabetes-induced cranial NTDs in rats, possibly through overcoming inositol depletion related to hyperglycaemia (Reece et al.,^1997^; Khandelwal et al.,^1998^). Interestingly, folic acidhas also been found to reduce the frequency of diabetes-induced NTDs (Oyama et al.,^2009^). Thus, in different contexts NTDs may be preventable either by folic acid or by inositol, or by both. The current study suggests a further possible protective role for inositol supplementation, in ameliorating NTDs resulting from folate deficiency.
